# Facial expressions and emotional experience during social interaction in autism and schizophrenia

**DOI:** 10.1038/s41537-026-00768-5

**Published:** 2026-05-26

**Authors:** Vladimír Ivančík, Natália Čavojská, Alexandra Straková, Jakub Januška, Hana Celušáková, Ján Pečeňák, Anton Heretik, Michal Hajdúk

**Affiliations:** 1 Department of Psychology, Faculty of Arts, Comenius University in Bratislava, Bratislava, Slovakia; 2 Centre for Psychiatric Disorders Research, Comenius University in Bratislava, Bratislava, Slovakia; 3 Department of Psychiatry, Faculty of Medicine, Comenius University in Bratislava, Bratislava, Slovakia; 4 Psychiatric Clinic, Slovak Medical University and University Hospital Bratislava, Bratislava, Slovakia

**Keywords:** Schizophrenia, Human behaviour

## Abstract

Blunted affect is a transdiagnostic feature that impairs social functioning across psychiatric conditions, including schizophrenia and autism. We quantified facial expression patterns and subjective emotional experience during standardized social interaction in 38 individuals with schizophrenia, 16 autistic adults, and 39 neurotypical adults using automated facial expression analysis. During social interaction, individuals with schizophrenia displayed more neutral expressions and reduced valence and arousal compared to neurotypical adults, whereas autistic adults showed typical facial expressions but subjectively experienced lower positive affect. These findings highlight distinct emotional profiles in schizophrenia and autism within social interactions, with blunted facial affect characterizing schizophrenia and reduced subjective positive affect characterizing autism.

## Introduction

Blunted affect is one of the most prominent and disabling negative symptoms of schizophrenia, strongly associated with impaired social functioning^1–4^. Similar reductions in emotional expressivity are also observed in autism spectrum disorders, where they form part of broader deficits in social communication and interaction^5^. These overlaps may complicate the interpretation of these symptom domains when considered in isolation, particularly at the level of observable social-communicative and affective expression, and may also pose challenges in distinguishing autism spectrum disorder from schizophrenia spectrum conditions based on current clinical presentation alone. This issue may be particularly relevant in adult clinical assessments of autism spectrum disorder, where early developmental history is sometimes limited or unavailable, making differential interpretation more challenging. Importantly, this distinction has significant clinical implications, as schizophrenia spectrum conditions typically warrant treatment with antipsychotic medication despite associated risks such as metabolic syndrome and tardive dyskinesia, whereas autism spectrum disorder alone does not necessarily require such intervention. Misclassification may therefore lead to unnecessary exposure to antipsychotic treatment in individuals with autism without comorbid schizophrenia, thereby highlighting the need to examine both shared and disorder-specific mechanisms underlying blunted affect^6–8^.

Within the Research Domain Criteria (RDoC) framework, blunted affect can be conceptualized as a dimensional construct reflecting disruptions in observable behavior that may be partially dissociated from one’s own subjective emotional experience^9,10^. Automated computer-based facial analysis now allows objective quantification of emotional expressions by tracking facial muscle movements, capturing frequency, intensity, and valence of affective displays^11^. Using these methods, reduced facial expressivity has been demonstrated in schizophrenia^12,13^ and in individuals at ultra-high risk for psychosis^13,14^, as well as in autism, where reduced positive expressions and increased neutral affect are evident from early development^15,16^. Despite evidence for blunted affect in both conditions, few studies have directly compared facial expression patterns and subjective emotional experience during live social interaction across schizophrenia, autism, and neurotypical adults using objective measures. Furthermore, examining associations between facial expression patterns and symptom severity is important for establishing the clinical relevance of objective affective measures in relation to core dimensions of psychopathology. Addressing this gap is essential to clarify transdiagnostic mechanisms and to identify disorder-specific profiles within RDoC-relevant domains.

Therefore, the present study aimed to: (1) identify patterns of facial expressions and subjective emotional experience during social interaction among individuals with schizophrenia, autism, and neurotypical adults, with the a priori expectation of increased neutral facial expressions in both clinical groups compared to neurotypical adults; (2) examine relationships between facial expressions and subjective emotional experience in both clinical groups; and (3) explore associations between facial expression patterns and symptom severity. Aims (2) and (3) are exploratory.

## Methods

### Participants

The sample consisted of 38 individuals with schizophrenia spectrum disorders (including schizophrenia and schizoaffective disorder, 55% female, M_age_ = 41.84, SD = 9.32), 16 individuals with autism spectrum disorders (44% female, M_age_ = 24.63, SD = 4.82), and 39 neurotypical adults (64% female, M_age_ = 35.41, SD = 10.40). The groups were matched on years of education. Individuals with autism were significantly younger than both individuals with schizophrenia and neurotypical adults, and neurotypical adults were younger than individuals with schizophrenia. Inclusion criteria were an IQ > 70 (based on clinical assessment) and sufficiently developed verbal abilities. General exclusion criteria included severe untreated somatic or neurological disorders, uncorrected visual impairment, history of serious head injury or epileptic seizures, as well as moderate to severe alcohol use disorder. In the schizophrenia group, the clinical diagnosis was confirmed by trained researchers utilizing Mini International Neuropsychiatric Interview (M.I.N.I.)^17^ for the schizophrenia group and Autism Diagnostic Observation Schedule – ADOS-2^18^ for the autism group. Individuals with comorbid schizophrenia spectrum disorder and autism spectrum disorder were not included in the sample. All participants provided voluntary informed consent. The study was approved by the Ethical Review Board of University Hospital Bratislava and the Medical Faculty of Comenius University in Bratislava.

### Measures

Computerized facial expression assessment was carried out in Noldus FaceReader version 9.1^19^. FaceReader is an automated facial expression analysis program that captures dynamic changes in facial expressions based on the Universal Facial Expressions of Emotion^20^. Participants completed a 3–minute unstructured social interaction with a same–sex research assistant, based on the Conversation Probe^21–23^. The interaction was framed as a scenario of getting to know a classmate at a language course, with the aim of approximating real-life social interaction as closely as possible under controlled experimental settings. Videotaped facial expressions of anger, disgust, fear, happiness, sadness, surprise, and neutral affect were analyzed using FaceReader. The software calculated the overall valence (ranging from positive to negative values) and arousal/intensity of the expressed emotions during the selected time window. The variables of interest were mean valence (index of emotional positivity vs. negativity, computed as the difference between happiness intensity and aggregated negative emotion intensity [anger, sadness, fear, and disgust]) and arousal (mean intensity of the most strongly activated facial muscle movements) of facial expressions, as well as mean time spent expressing a neutral face (absence of detectable basic emotion expressions), which was also treated as a continuous output from FaceReader. Mean scores of neutral affect and arousal were computed on a scale ranging from 0 to 1, while the mean valence was scaled from -1 (negative) to 1 (positive). All variables were averaged over the full 3-minute interaction window based on frame-level data down-sampled at 5 frames per second. This dimensional approach was selected a priori to provide a parsimonious characterization of emotional expressions while reducing the number of statistical comparisons associated with analyzing multiple discrete emotion categories. While prior literature often reports discrete emotional expressions, these can be conceptually mapped onto the valence dimension.

Subjective emotional experience after completing the social interaction was measured by the Positive and Negative Affect Schedule–10 (PANAS-10)^24^. Participants rated their current positive and negative affect on a 5–point Likert scale.

Symptom severity in the clinical groups was rated by an independent research assistant using the Brief Psychiatric Rating Scale (BPRS). A four-factor solution was used to classify symptoms into domains: positive, negative, agitation/mania, and depression/anxiety^25^.

All participants completed the Community Assessment of Psychic Functioning (CAPE-42)^26^ to assess the frequency of positive, negative, and depressive symptoms. In addition, participants from all groups were also administered the Comprehensive Autistic Trait Inventory (CATI)^27^ as an indicator of autistic traits. The total score was used to quantify the symptom severity.

### Statistical analysis

Group differences in facial expressions and subjective emotional experiences were examined using a series of ANOVAs with subsequent post-hoc tests with Bonferroni correction. Spearman's rank correlations were utilized to explore the relationships between facial expressions and symptom severity for each clinical group. Additionally, Spearman's rank correlations were conducted to explore the relationships between facial expressions and subjective emotional experience for all groups. The correlation analyses were conducted in an exploratory framework; therefore, no correction for multiple comparisons was applied.

### Ethics approval and consent to participate

The study was reviewed and approved by the ethical committees at the Faculty of Arts, Comenius University, and the University Hospital Bratislava. This study has been performed in accordance with the ethical standards laid down in the 1964 Declaration of Helsinki and its later amendments.

## Results

### Descriptive statistics

Table 1 summarizes the demographic and clinical characteristics of the sample. Clinical groups did not differ in clinician-rated symptom severity assessed by the BPRS. However, both clinical groups reported more severe positive as well as negative symptoms (CAPE-42) when compared to neurotypical adults. For autistic traits (CATI), we found group differences, as shown in Table 1.

**Table 1:** Demographic and clinical characteristics.

	SCH M (SD)	ASD M (SD)	HC M (SD)	Difference test	Post-hoc analysis (Bonferroni corrected)
*N* (% females)	38 (55%)	16 (44%)	39 (64%)		
Age	41.84 (9.32)	24.63 (4.82)	35.41 (10.40)	F (2, 90) = 19.82 *p* < 0.001	SCH > HC > ASD
Years of education	15.55 (3.08)	15.06 (3.02)	16.85 (3.86)	F (2, 90) = 2.118 *p* = 0.126	–
Positive symptoms – BPRS	12.50 (6.29)	10.38 (3.65)		U = 349.500 *p* = 0.390	–
Negative symptoms – BPRS	6.97 (3.04)	7.25 (2.65)		U = 276.000 *p* = 0.599	–
Agitation/ mania – BPRS	8.45 (4.20)	9.19 (2.86)		213.500 *p* = 0.079	–
Depression/ Anxiety – BPRS	9.26 (4.51)	11.13 (4.53)		U = 225.500 *p* = 0.138	–
Autistic traits – CATI	122.57 (19.52)	159.50 (19.09)	102.89 (19.52)	F (2, 86) = 43.424 *p* < 0.001	ASD > SCH > HC
Positive symptoms – CAPE-42	30.08 (7.37)	29.00 (7.13)	24.03 (3.51)	F (2, 88) = 10.484 *p* < 0.001	SCH, ASD > HC
Negative symptoms – CAPE-42	27.45 (7.75)	29.73 (3.77)	23.90 (5.34)	F (2, 89) = 5.746 *p* = 0.004	SCH, ASD > HC

*SCH* individuals with schizophrenia, *ASD* individuals with autism, *HC* neurotypical adults.

### Facial expression patterns across groups

Significant group differences were found in overall valence of facial expressions (F (2, 75) = 5.765, *p* = 0.005, ω^2^ = 0.109), with individuals with schizophrenia having overall lower valence compared to neurotypical adults (see Table 2). In addition, we found different patterns in arousal of expressed emotions between groups (F (2, 75) = 6.895, *p* = 0.002, ω^2^ = 0.131). Individuals with schizophrenia expressed emotions less intensively compared to neurotypical adults. Group differences were also present in neutral expressions (F (2, 75) = 4.097, *p* = 0.020, ω^2^ = 0.074), where individuals with schizophrenia exhibited increased neutral expressions relative to neurotypical adults. No additional significant group differences in facial expression patterns were observed. Emotion-specific facial expressions were explored as a Supplementary analysis.

**Table 2:** Facial expressions and subjective emotional experience.

		HC M (SD)	SCH M (SD)	ASD M (SD)	Post-hoc analysis (Bonferroni corrected)
Facial expressions	Valence	0.227 (0.263)	−0.006 (0.271)	0.137 (0.289)	HC > SCH
	Arousal	0.367 (0.041)	0.330 (0.035)	0.341 (0.045)	HC > SCH
	Neutral expression	0.462 (0.223)	0.613 (0.200)	0.490 (0.235)	SCH > HC
	Positive affect	16.769 (3.166)	14.778 (5.238)		
Subjective emotional experience				13.625 (4.064)	HC > ASD
	Negative affect	7.564 (3.447)	8.457 (3.081)	9.625 (4.080)	–

*SCH* individuals with schizophrenia, *ASD* individuals with autism, *HC* neurotypical adults.

### Subjective emotional experience across groups

The differences in subjective emotional experience are shown in Table 2. The groups differed in subjectively experienced positive affect (F (2, 88) = 3.805, *p* = 0.026, ω^2^ = 0.058), which was significantly lower in individuals with autism, compared to neurotypical adults. No significant differences were found in subjective emotional experience between autism and schizophrenia. No significant group differences were found in the subjective emotional experience of negative affect.

### Associations between facial expression patterns and symptoms severity – exploratory analysis

In schizophrenia, higher arousal of facial expressions was linked to less severe BPRS-rated agitation/mania symptoms (r_s_ = −0.362, *p* = 0.046). There were no significant correlations between facial expression patterns and self-reported psychotic symptoms (CAPE-42) or autistic traits (CATI) in schizophrenia. We found no relationships between symptom severity (both clinician-rated and self-reported) and facial expression patterns in the autism group.

### Associations between facial expression patterns and subjective emotional experience – exploratory analysis

We found no relationships between facial expressions and subjective emotional experience in individuals with schizophrenia. In the autism group, greater positive affect was positively associated with valence (r_s_ = 0.524, p = 0.037) and arousal (r_s_ = 0.500, *p* = 0.049).

## Discussion

The current study investigated facial expression patterns and subjective emotional experience during social interaction in individuals with autism and schizophrenia. To our knowledge, this is the first study to directly compare these domains in both clinical groups using an automated computer-based analysis.

Our findings revealed that individuals with schizophrenia exhibited significantly reduced facial expressivity, as evidenced by more frequent neutral expressions, and overall lower valence of facial expressions, when compared to neurotypical adults. These results align with previous research suggesting that blunted affect is a prominent symptom of schizophrenia^12,13^. Interestingly, individuals with autism did not differ significantly from either schizophrenia or neurotypical adults in terms of facial expression patterns. This finding is surprising considering prior evidence suggesting decreased emotional expressions in autism compared to the general population^5^. This may reflect the inclusion of relatively high-functioning individuals in our sample. Individuals with autism reported experiencing lower levels of positive affect after social interaction compared to neurotypical adults, consistent with prior findings of reduced subjective positive emotional experiences in autism^28^.

A key difference between the two clinical groups was found in the relationship between subjective emotional experience and objectively measured facial expression patterns. In schizophrenia, facial expressions were not associated with reported subjective emotion experiences, supporting existing evidence that emotional blunting in schizophrenia may reflect a disconnection between internal experience and outward expression of the given experience^9^. In contrast, autistic individuals who reported experiencing more positive emotions during the interaction also expressed emotions with overall higher valence and arousal, suggesting greater congruence between emotional experience and expression.

Associations between facial expressions and symptom severity were only observed in the schizophrenia group, where greater emotional expressivity was linked to lower levels of agitation/mania. While this pattern may tentatively reflect differences in affect regulation processes in autism and schizophrenia, the small sample size and the exploratory nature of the analyses warrant caution in interpretation, as the finding may represent a false positive association rather than a robust effect. No such associations were observed in the autism group, although limited statistical power may have constrained the detection of potential effects.

The present study has several limitations. First, the relatively small sample size, particularly within the subgroup of individuals with autism, may have limited statistical power and may not have revealed some important associations or may have potentially skewed the results. Furthermore, the analyses examining associations between facial expression indices and subjective emotional experience, as well as those exploring relationships with symptom severity, were purely exploratory and should therefore be interpreted accordingly with caution. Given the small sample size of the study, we did not adjust for potential confounding variables such as age, sex, or medication status, which may have potentially biased the results. In addition, autism spectrum disorders encompass individuals with a wide range of impairments in social functioning, verbal abilities, and intellectual difficulties. Therefore, the characteristics of the selected sample may introduce potential biases that could affect the generalization of findings. To more accurately capture the heterogeneity of clinical manifestation of autism as reflected in facial expressivity, future research should aim to include individuals with more severe autistic symptoms. Finally, social interactions during the experimental task were conducted by neurotypical researchers who were not blinded to participants’ clinical diagnoses and were instructed to behave in a friendly manner. This may have reduced the ecological validity of the social interaction task.

Overall, these findings support the utility of automated facial expression analysis as a tool for studying transdiagnostic features of blunted affect and highlight important distinctions in emotional expressions and experience in autism and schizophrenia. Importantly, these methods provide a more fine-grained and objective assessment of affective behavior, capturing subtle and dynamic changes in emotional expression during social interaction that may not be readily observable even to experienced clinicians, and allowing for the detection of subtle fluctuations in affective expressivity that may be missed by traditional assessment approaches.

Beyond diagnostic considerations, these findings have implications for understanding social functioning in schizophrenia and autism. Difficulties in translating subjective emotional states into appropriate facial expressions may contribute not only to interpersonal misunderstandings but also to reduced willingness of others to engage in social interaction, thereby further limiting opportunities for social connection^29^. In turn, impaired social functioning is closely linked to poor real-world outcomes in both conditions, including reduced social support, employment difficulties, and increased vulnerability to social marginalization. Understanding these processes may help inform interventions targeting emotional communication and social functioning in autism and schizophrenia.

## Supplementary information

Supplement

## Data Availability

The dataset is available from the corresponding author upon a reasonable request.
