# Supplementary material for: Facial expressions and emotional experience during social interaction in autism and schizophrenia
Source: Schizophrenia (Heidelb). 2026 May 26;12(1):68. doi: 10.1038/s41537-026-00768-5 (PMC13486720; doi:10.1038/s41537-026-00768-5)
Supplement: Supplementary file 1 [file 41537_2026_768_MOESM1_ESM.docx]

**Supplementary information**

**Supplementary analysis 1 – Emotion-specific facial expressions**

Group differences in emotion-specific facial expressions during social interaction were explored; results are shown in Supplementary Table 1. The groups differed in expressed happiness (F (2, 75) = 5.933, p = 0.004, ω^2^ = 0.112), with neurotypical adults exhibiting increased expressions of happiness compared to individuals with schizophrenia. Additionally, group differences were found in expression of anger (F (2, 75) = 3.655, p = 0.031, ω^2^ = 0.064), with individuals with autism expressing more anger compared to neurotypical adults. There were no group differences in expressed sadness, surprise, fear, or disgust.

**Supplementary Table 1** Emotion-specific facial expressions

| Emotion | HC  M (SD) | SCH  M (SD) | ASD  M (SD) | Post-hoc (Bonferroni corrected) |
| --- | --- | --- | --- | --- |
| Happiness | 0.227  (0.263) | 0.314  (0.228) | 0.122  (0.203) | HC > ASD |
| Sadness | 0.054  (0.059) | 0.057  (0.078) | 0.044  (0.057) | – |
| Anger | 0.022  (0.043) | 0.046  (0.067) | 0.074  (0.086) | ASD > HC |
| Surprise | 0.070  (0.074) | 0.037  (0.062) | 0.071  (0.105) | – |
| Fear | 0.013  (0.019) | 0.012  (0.026) | 0.019  (0.030) | – |
| Disgust | 0.031  (0.079) | 0.059  (0.106) | 0.019  (0.014) | – |

*SCH – individuals with schizophrenia; ASD – individuals with autism; HC – neurotypical adults*
